# Supplementary material for: Critical Role of Cathepsin L/V in Regulating Endothelial Cell Senescence
Source: Biology (Basel). 2022 Dec 26;12(1):42. doi: 10.3390/biology12010042 (PMC9855167; doi:10.3390/biology12010042)
Supplement: Supplementary file 1 [file biology-12-00042-s001.zip › Supplementary materials.pdf]

Supplementary Materials

Table S1. Primer sequences for qRT-PCR.

Gene name	Forward primer(5'-3')	Reverse primer(5'-3')
Cathepsin V	TGGAAGGCAACACACAGAAG	GAAGCCATGTTTCCCTTGG
p53	GGCCCACTTCACCGTACTAA	GTGGTTTCAAGGCCAGATGT
p16	GACATCCCCGATTGAAAGAA	TTTACGGTAGTGGGGGAAGG
p21	GACACCACTGGAGGGTGACT	CAGGTCCACATGGTCTTCCT
ALDH1A2	GGATGACCATTCCTGTAGATGGAGA	GCTATTGCTGCCCCAGCCGTTG
THBD	GGAGCAGCAGTGCGAAGT	GTGGCTGGGAAGTGGAACT
IGFBP5	TGAGATGAGACAGGAGTCTGAG	GTCACAATTGGGCAGGTACA
APOLD1	AGCCAAGCAGAACACAGTCCAAG	CACTCCAGCCACAGCACTCATTC
NOS3	TCTCCGCCTCGCTCATG	AGCCATACAGGATTGTCGCC
LAMA5	CGAGGACCTTTACTGCAAGC	GGTGACGTTGACCTCGTTGT
SEMA3F	CGCGAGCCCCTCATTATACA	TGACGAAGTTCCCACACTCG
SLC9A3R2	GACCAGGAGACAGATGAGGAG	ACTGACATCCTTCTTGCCAG
IL-1β	ACAGATGAAGTGCTCCTTCCA	GTCGGAGATTCGTAGCTGGAT
IL-6	GCAGAAAACAACCTGAACCTT	ACCTCAAACTCCAAAAGACCA
ICAM-1	AGCGGCTGACGTGTGCAGTAAT	TCTGAGACCTCTGGCTTCGTCA
β-actin	TGGACTTCGAGCAAGAGATG	TGTTGGCGTACAGGTCTTTG

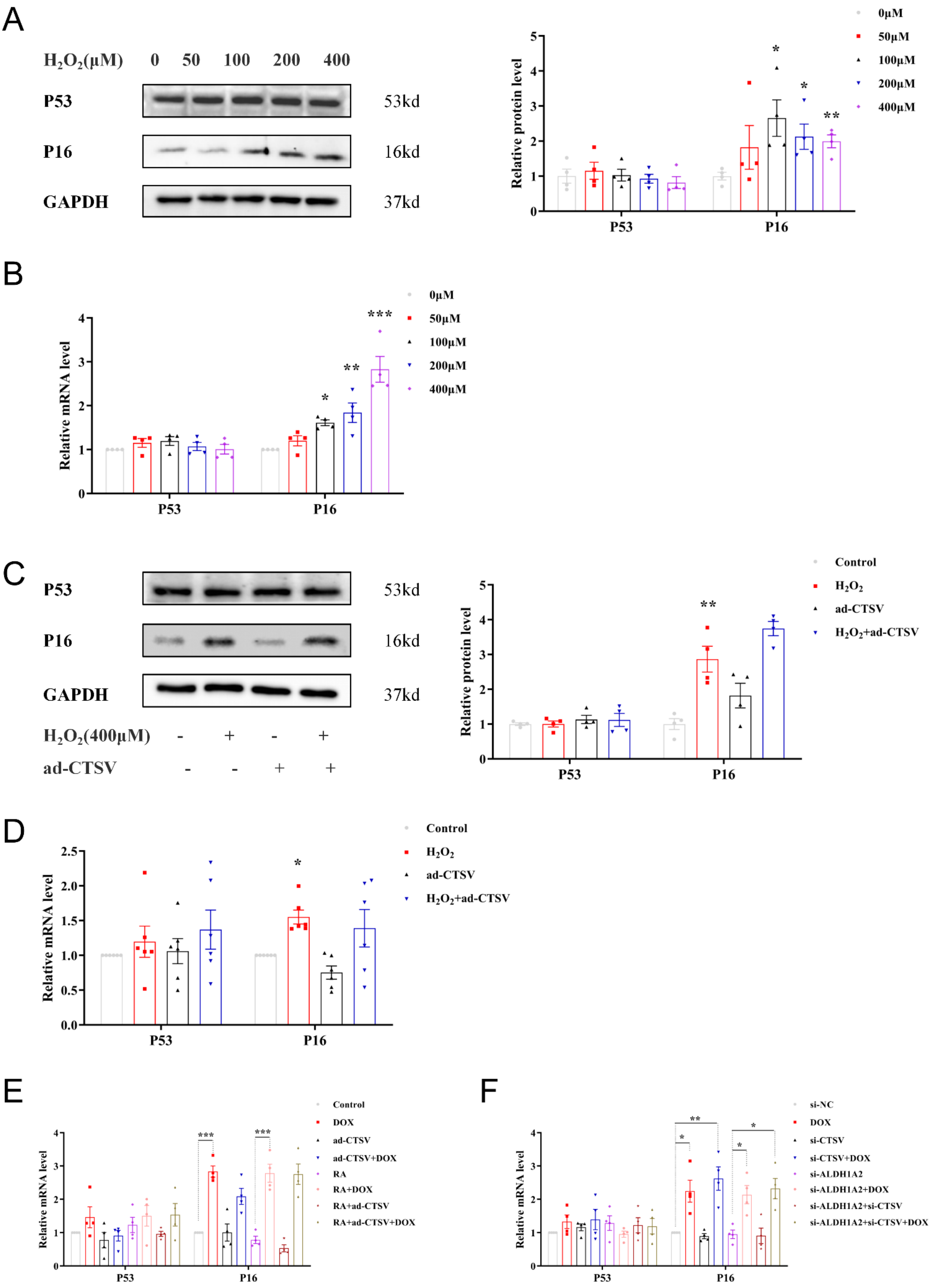
A
H2O2(μM) 0 50 100 200 400
P53
P16
GAPDH
53kd
16kd
37kd
Relative protein level
0μM
50μM
100μM
200μM
400μM
P53
P16
B
Relative mRNA level
0μM
50μM
100μM
200μM
400μM
P53
P16
C
P53
P16
GAPDH
53kd
16kd
37kd
H2O2(400μM) - + - +
ad-CTSV - - + +
Relative protein level
Control
H2O2
ad-CTSV
H2O2+ad-CTSV
P53
P16
D
Relative mRNA level
Control
H2O2
ad-CTSV
H2O2+ad-CTSV
P53
P16
E
Relative mRNA level
Control
DOX
ad-CTSV
ad-CTSV+DOX
RA
RA+DOX
RA+ad-CTSV
RA+ad-CTSV+DOX
P53
P16
F
Relative mRNA level
si-NC
DOX
si-CTSV
si-CTSV+DOX
si-ALDH1A2
si-ALDH1A2+DOX
si-ALDH1A2+si-CTSV
si-ALDH1A2+si-CTSV+DOX
P53
P16

Figure S1. Expression of P53 and P16 in HUVECs treated with H_2O_2. **A.** HUVECs were treated with different dose of H_2O_2 (0μM, 50μM, 100μM, 200μM, and 400μM) for 24 h, expression of P53 and P16 were tested by western blot ($n = 4$). **B.** Relative mRNA level of P53 and P16 in H_2O_2-treated HUVECs ($n = 4$). **C.** HUVECs were treated with ad-negative control (NC) or ad-CTSV (MOI = 10) for 8 h, and simulated with 400 μM H_2O_2 for 24 h after incubated in complete growth medium for 40 hours, expression of P53 and P16 were tested by western blot ($n = 4$). **D.** Relative mRNA level of P53 and P16 in HUVECs treated with H_2O_2 and ad-CTSV ($n = 6$). **E.** Relative mRNA level of P53 and P16 in HUVECs treated with DOX, ad-CTSV and RA ($n = 4$). **F.** Relative mRNA level of P53 and P16 in HUVECs treated with DOX, si-CTSV and si-ALDH1A2 ($n = 4$). Data were presented as mean ± SEM. One-way ANOVA test was used. * $p < 0.05$ vs. Control, ** $p < 0.01$ vs. Control, *** $p < 0.001$ vs. Control.

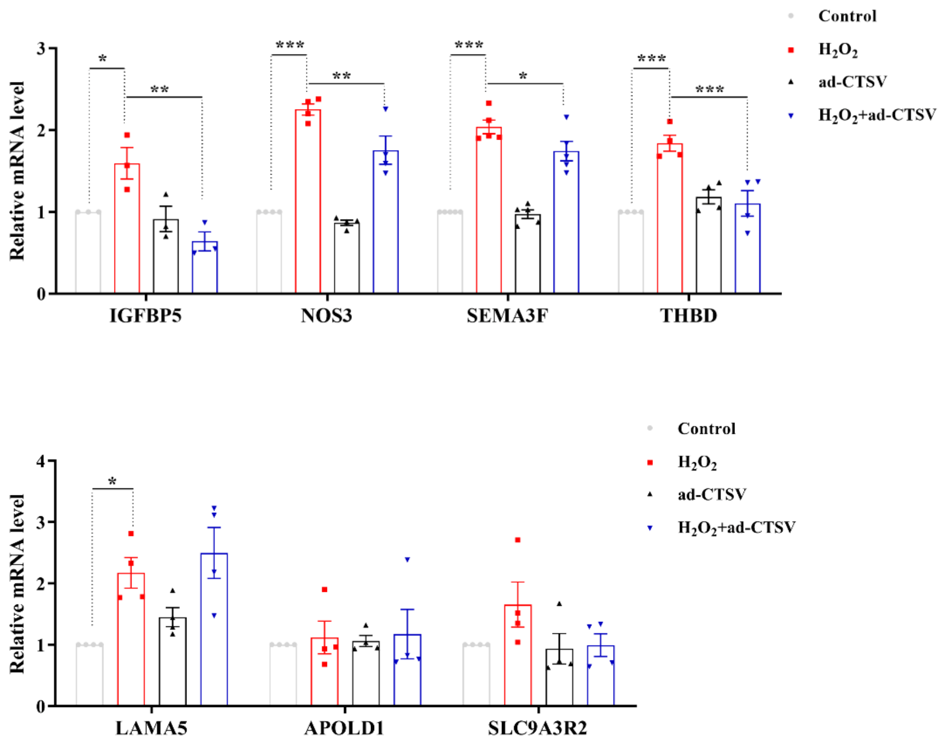

Figure S2. Relative mRNA level of the differentially expressed genes. HUVECs were treated with ad-negative control (NC) or ad-CTSV (MOI = 10) for 8 h, and simulated with 400 μM H_2O_2 for 24 h after incubated in complete growth medium for 40 h. Relative mRNA level of *IGFBP5*, *NOS3*, *THBD*, *SEMA3F*, *LAMA5*, *APOLD1*, and *SLC9A3R2* were presented ($n = 3$–5). Data were presented as mean ± SEM. One-way ANOVA test was used. * $p < 0.05$, ** $p < 0.01$, *** $p < 0.001$.

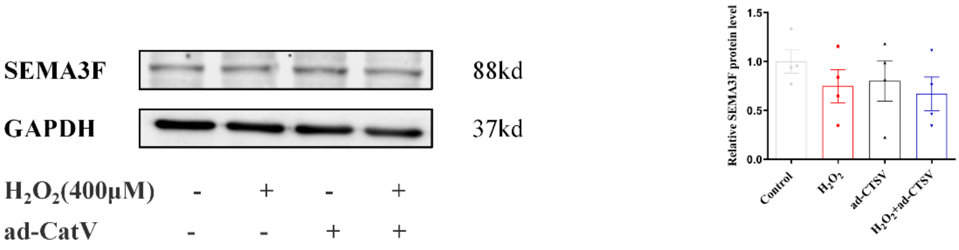

Figure S3. Protein level of SEMA3F. HUVECs were treated with ad-negative control (NC) or ad-CTSV (MOI = 10) for 8 h, and simulated with 400 μM H_2O_2 for 24 h after incubated in complete growth medium for 40 hours. Western blot of SEMA3F was presented ($n = 4$). Data were presented as mean ± SEM. One-way ANOVA test was used.

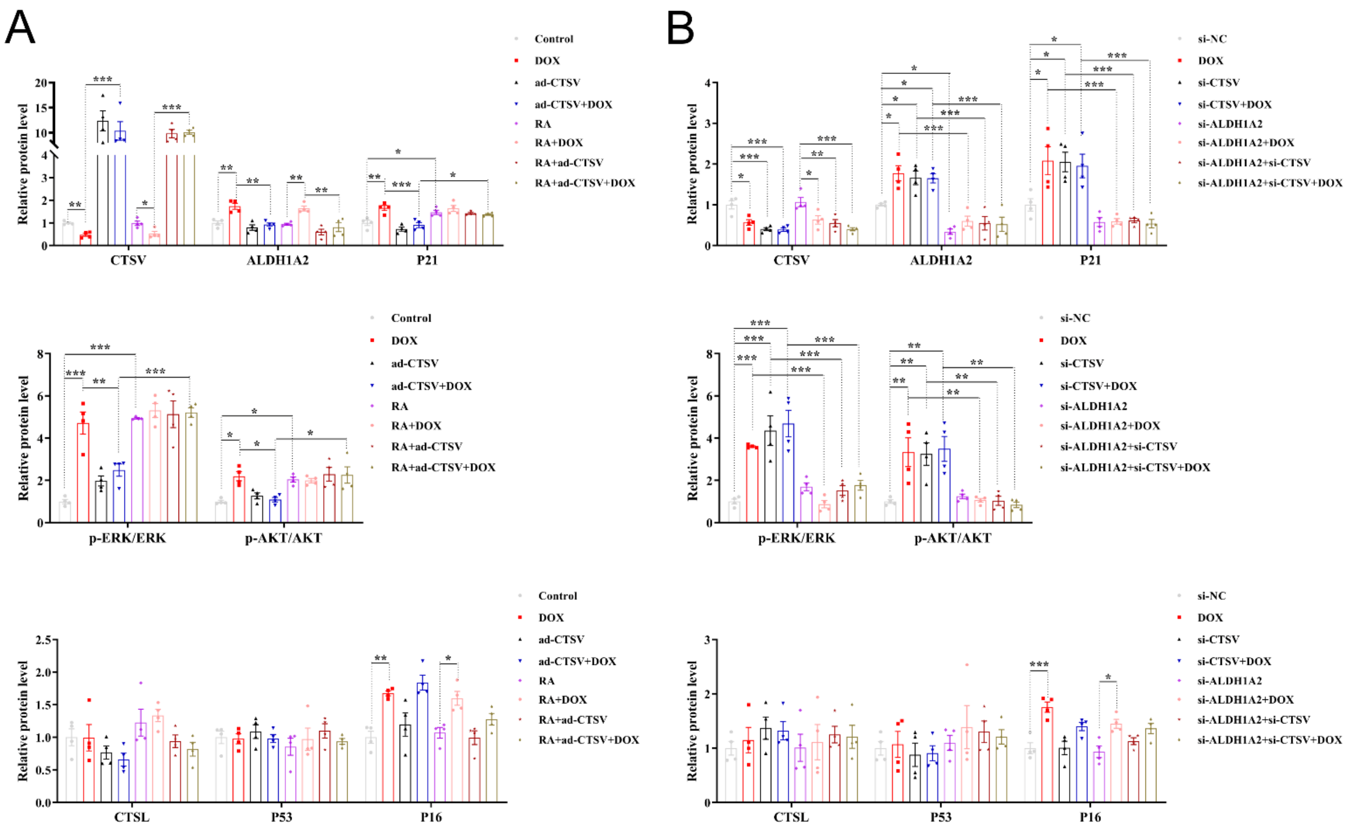

Figure S4. Quantification of Western Blots. **A.** Quantification of Figure 6E. **B**. Quantification of Figure 7E. Data were presented as mean ± SEM. One-way ANOVA test was used. * $p < 0.05$, ** $p < 0.01$, *** $p < 0.001$.
