# Supplementary figures and images for: Critical Role of Cathepsin L/V in Regulating Endothelial Cell Senescence
Source: Biology (Basel). 2022 Dec 26;12(1):42. doi: 10.3390/biology12010042 (PMC9855167; doi:10.3390/biology12010042)

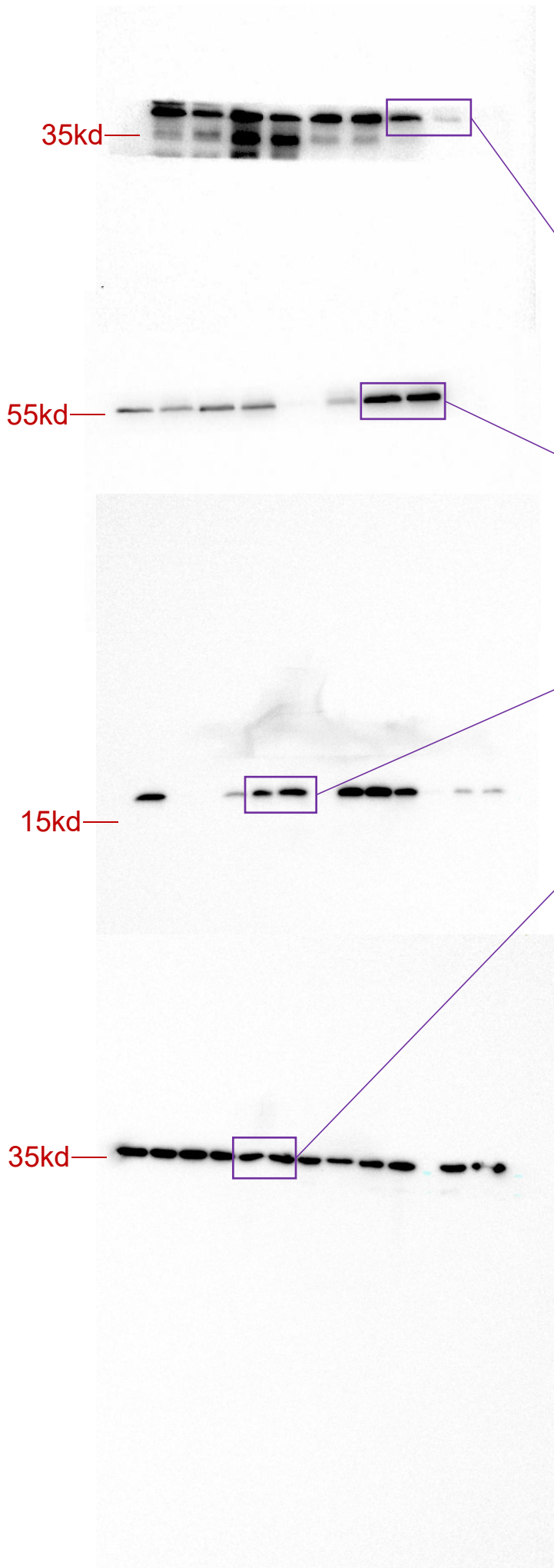

**Figure 1**

**E**

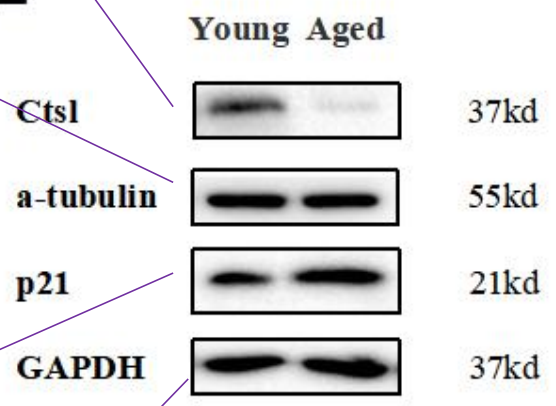

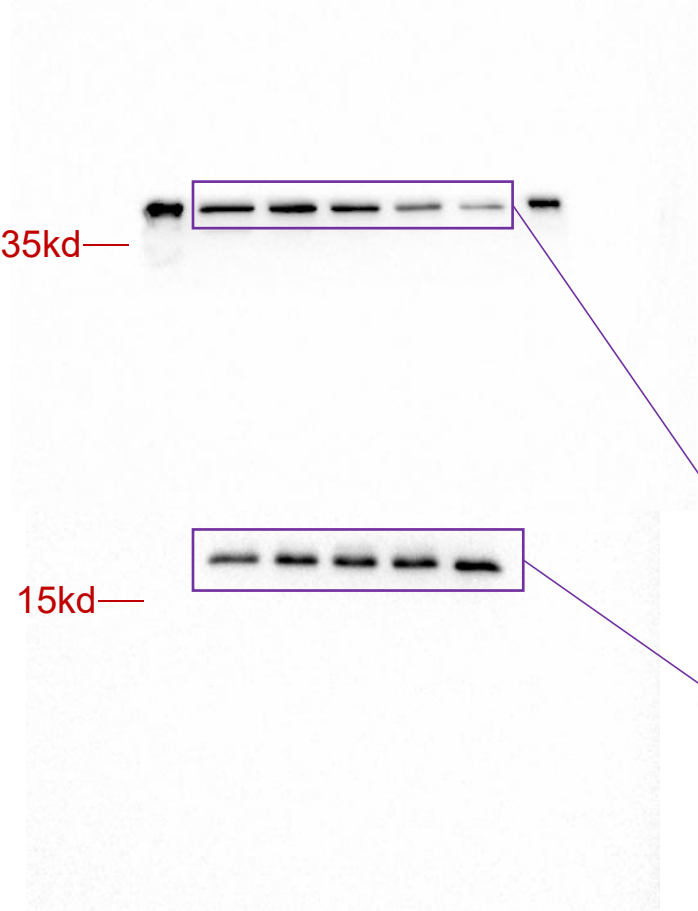

**Figure 2**

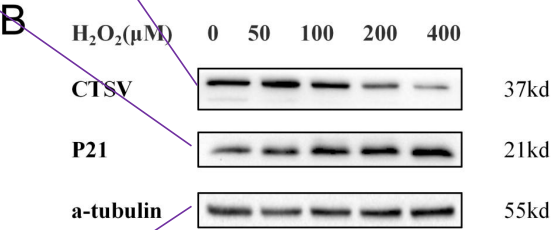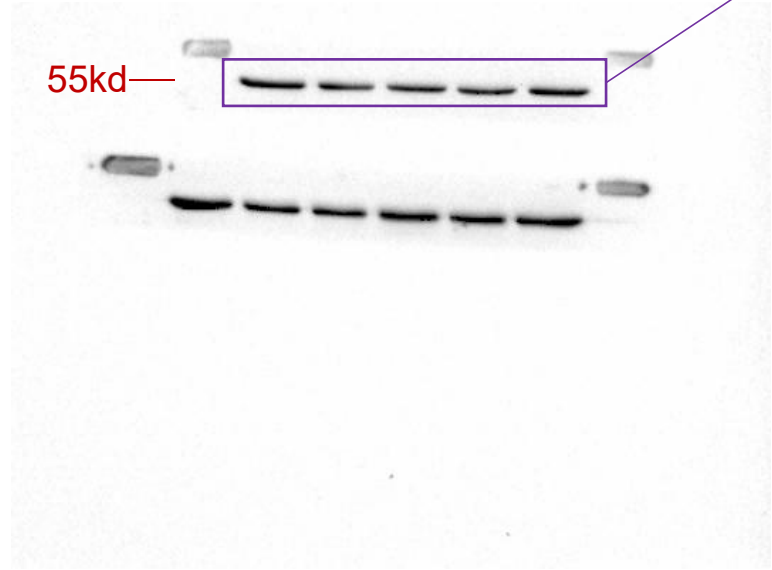

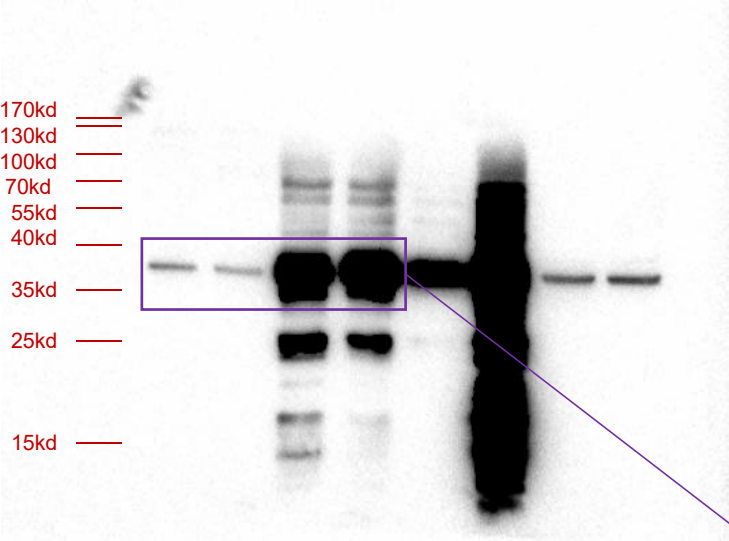

**Figure 3**

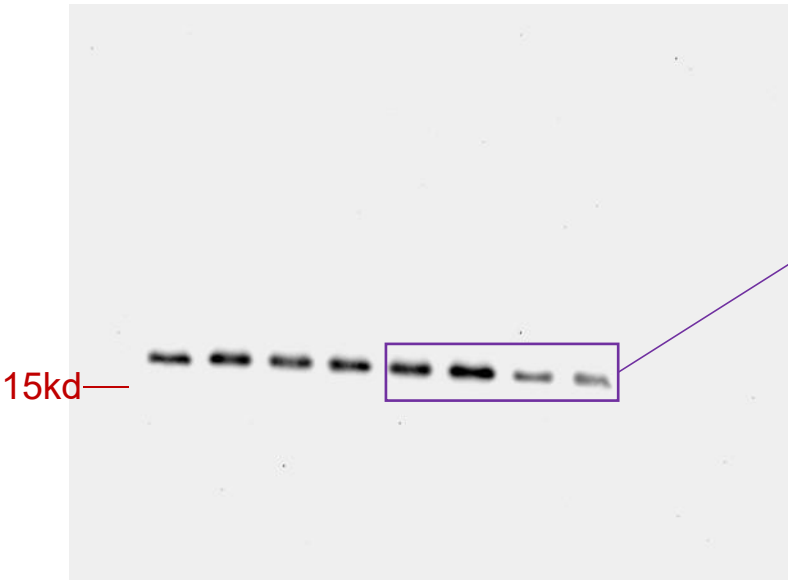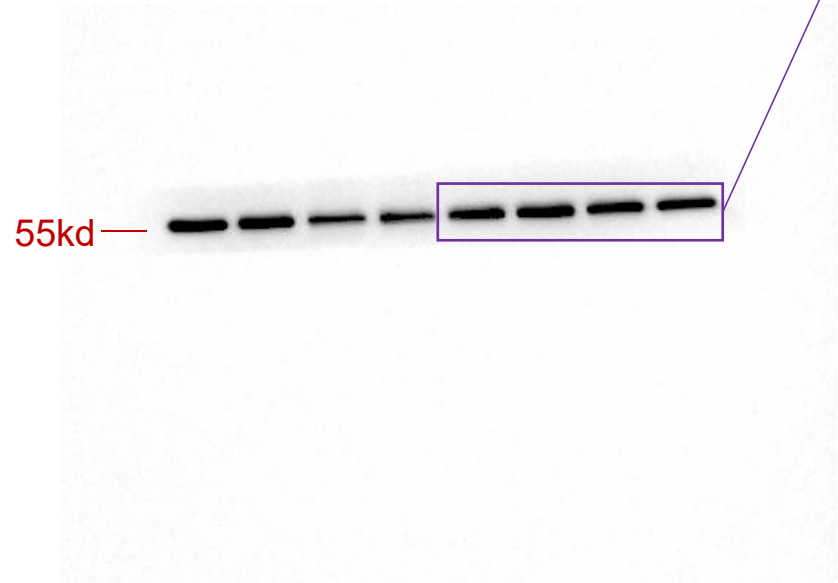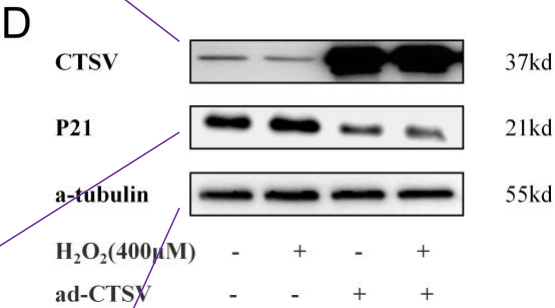

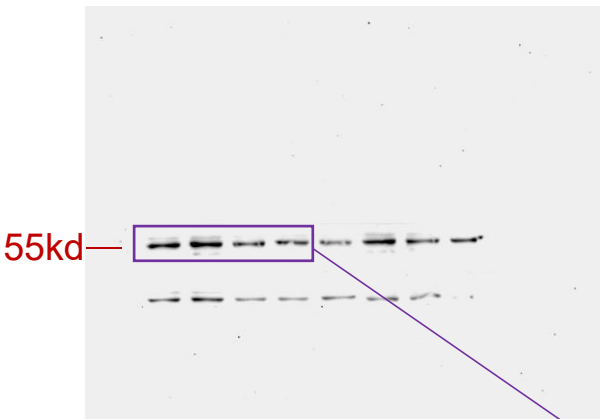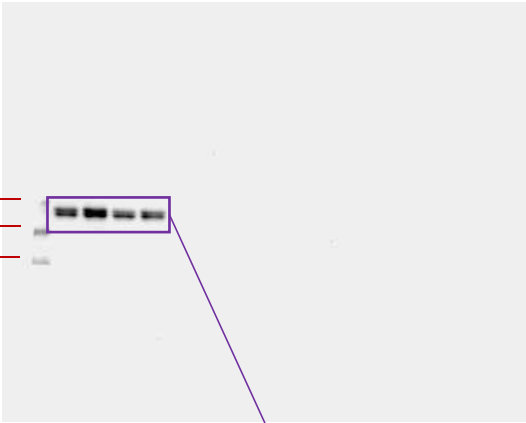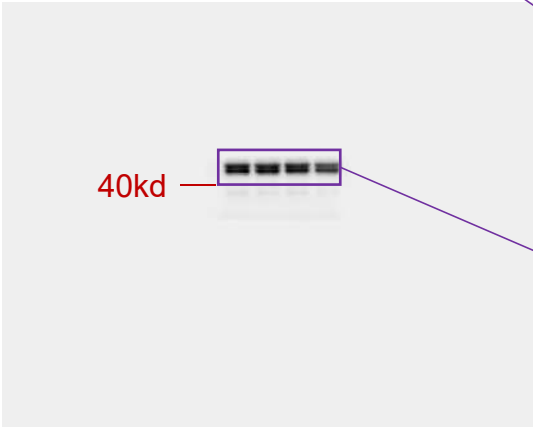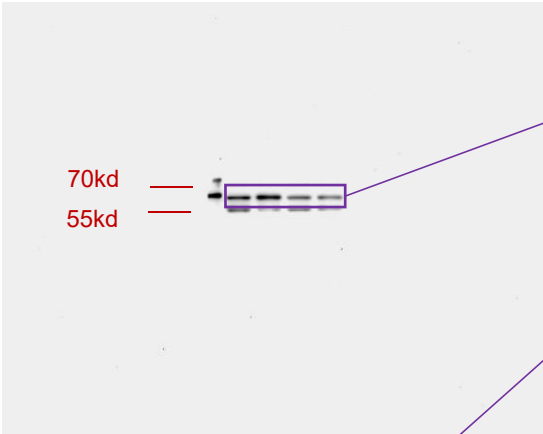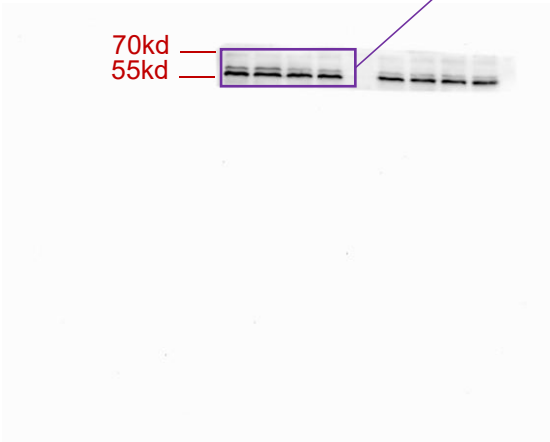

D

Figure 4

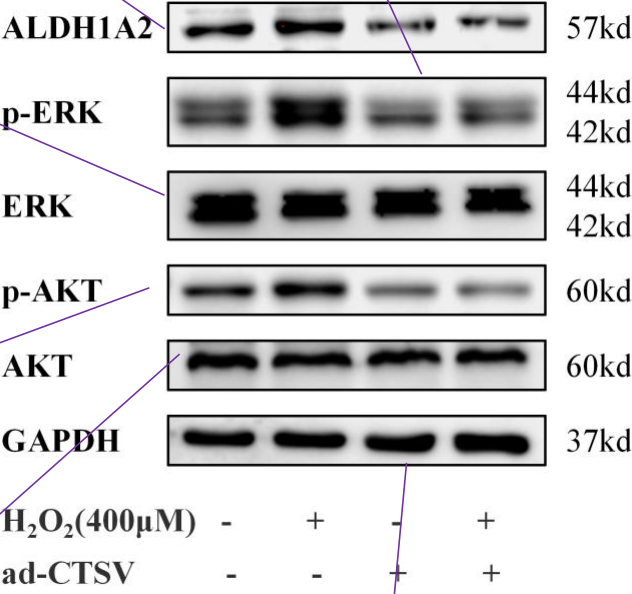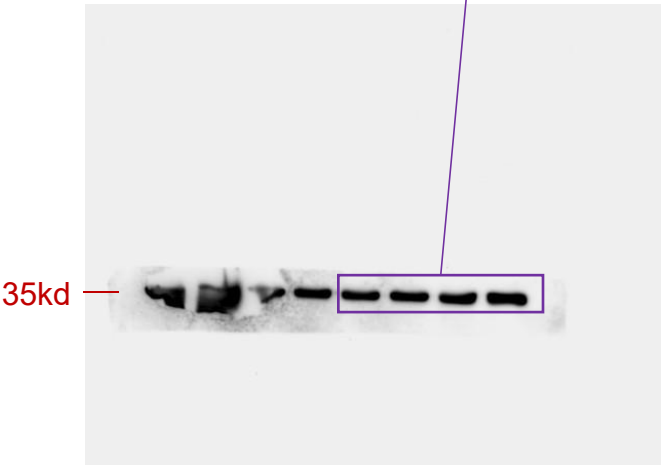

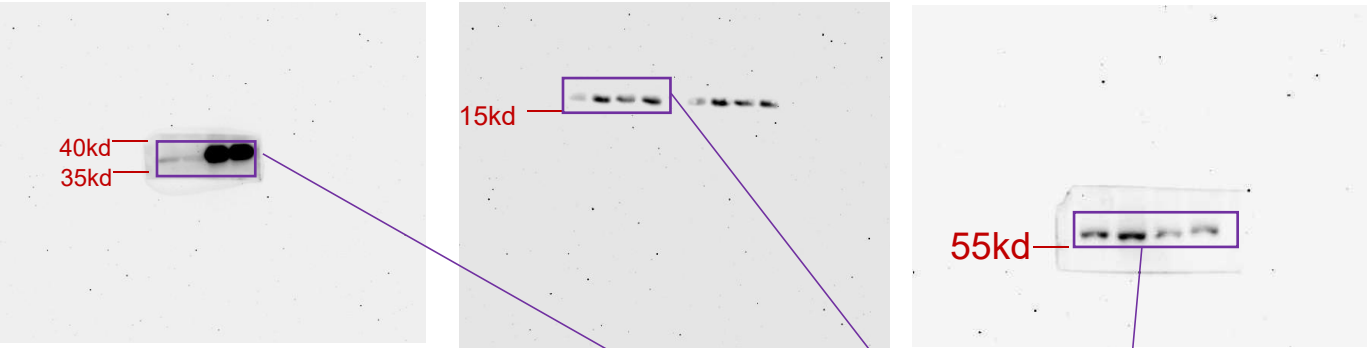

**Figure 5**

**D**

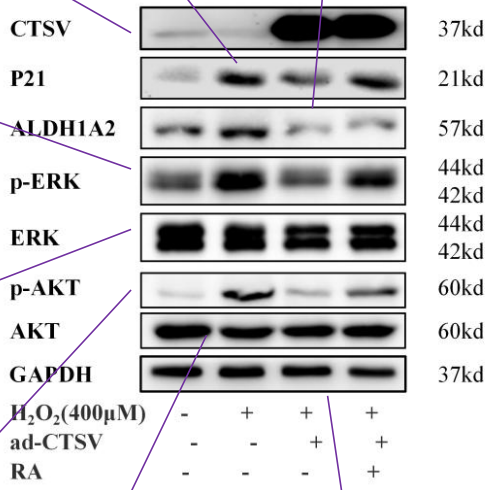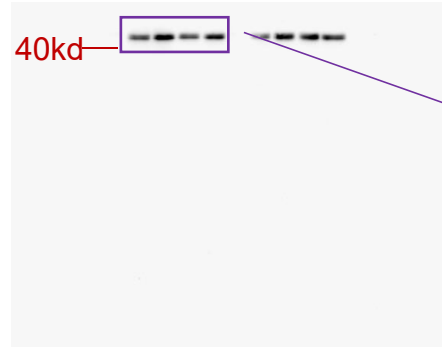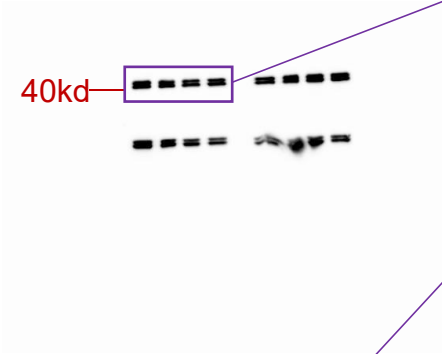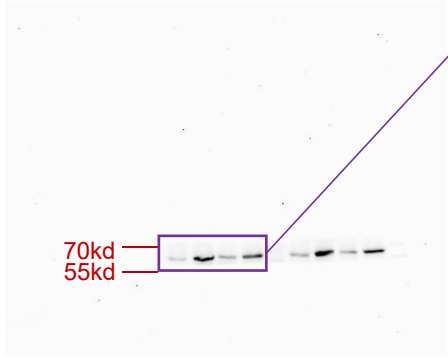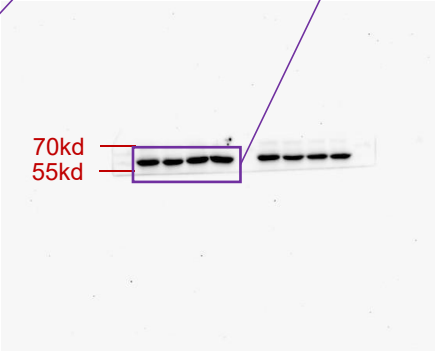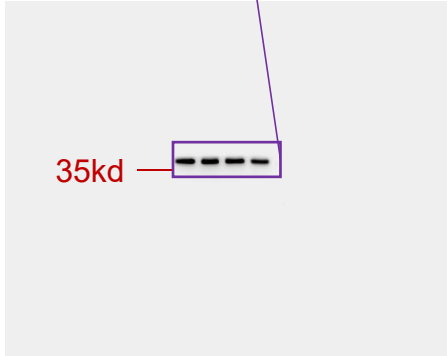

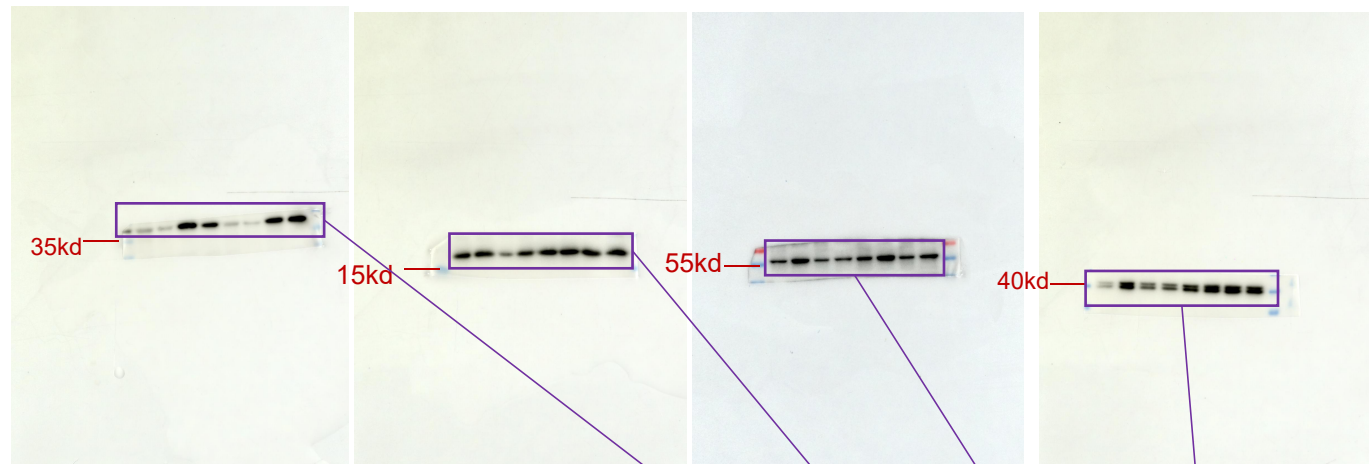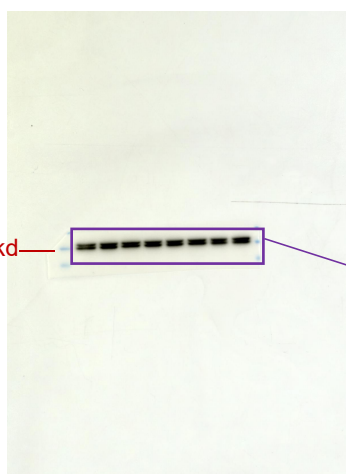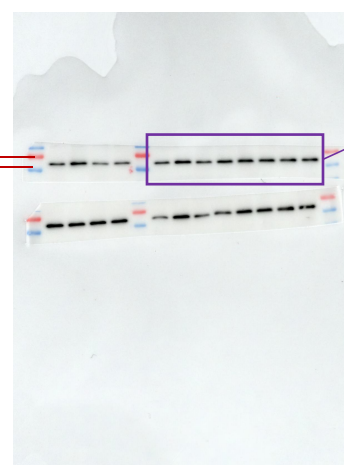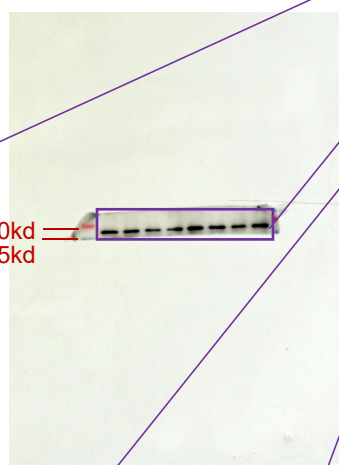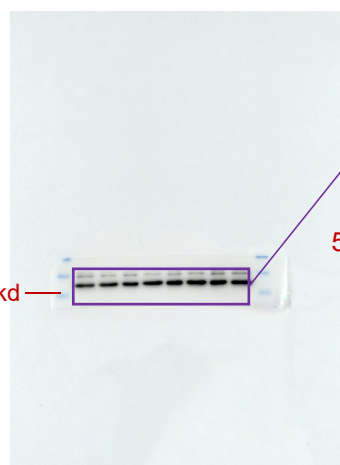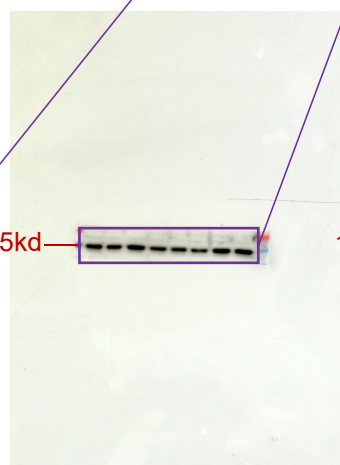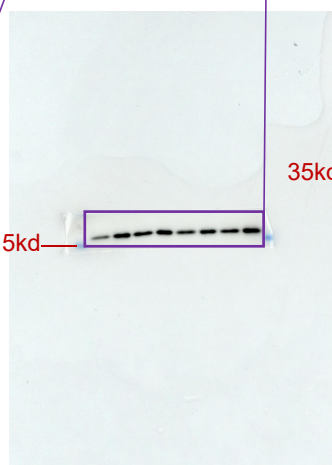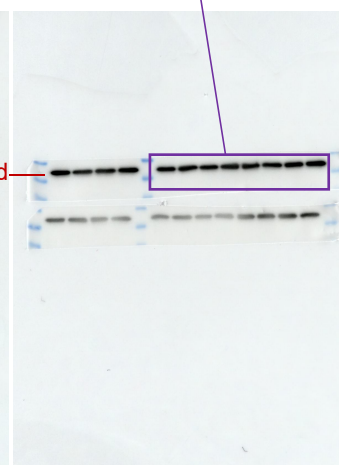

**Figure 6**

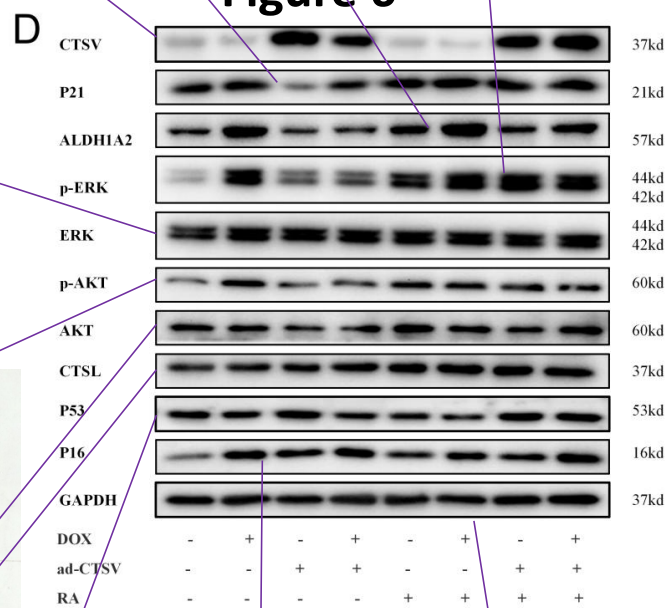

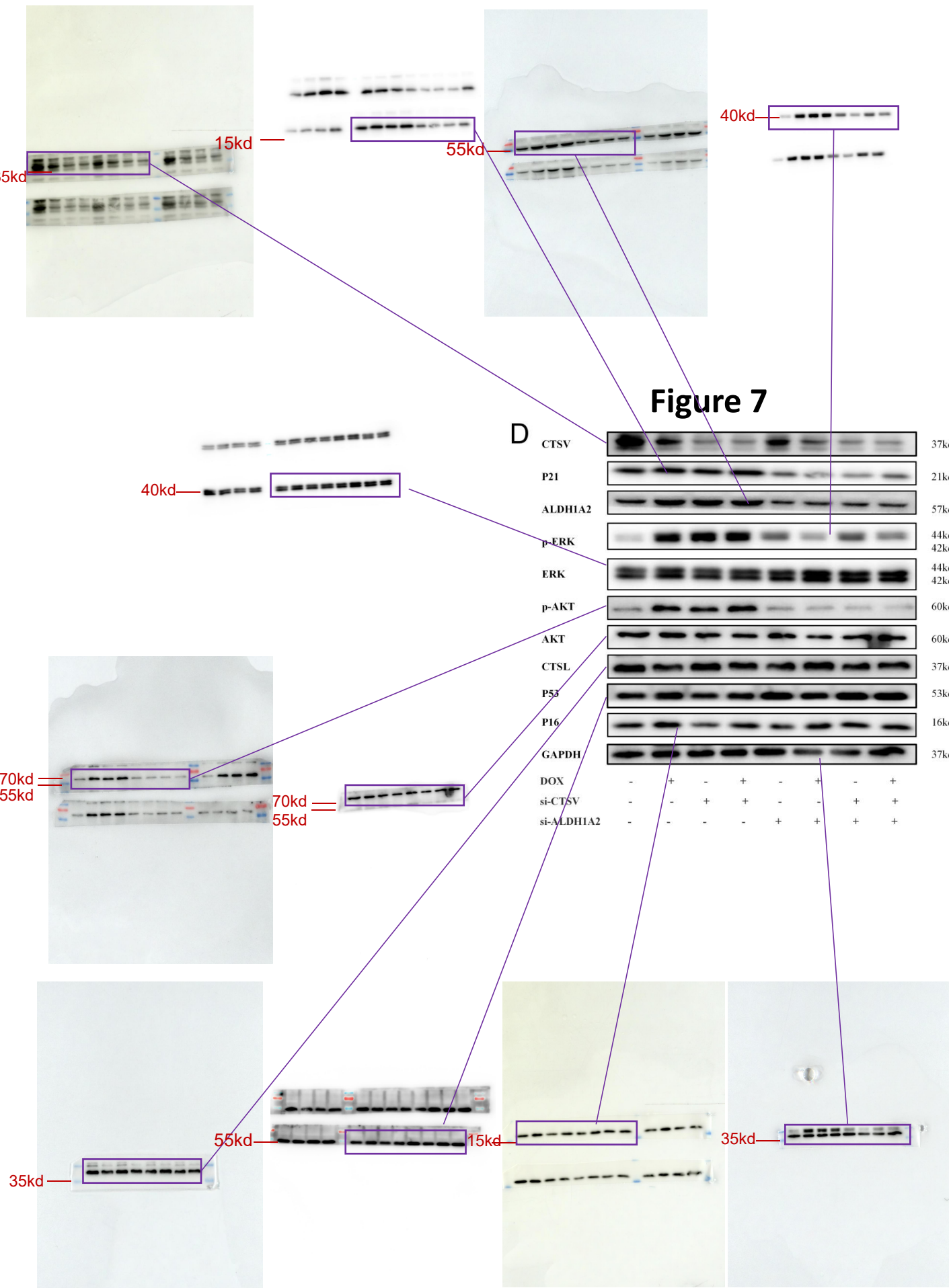

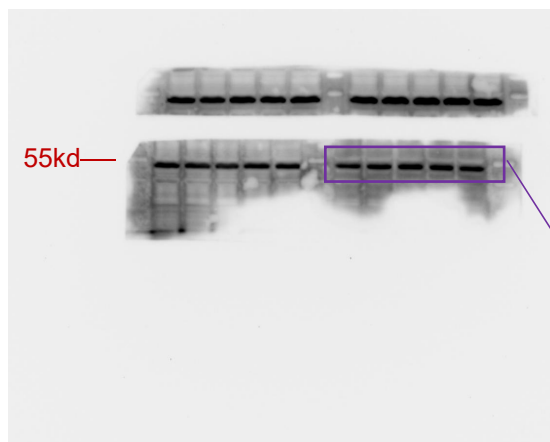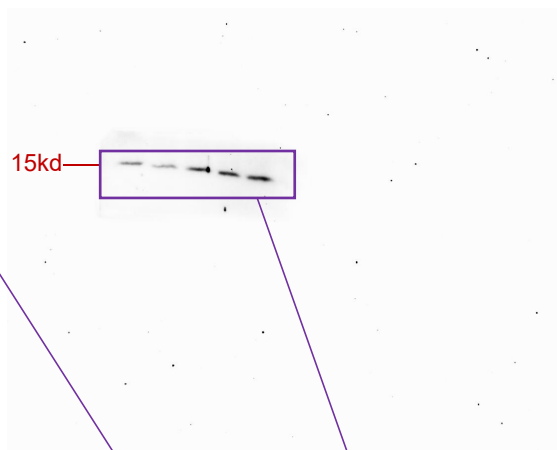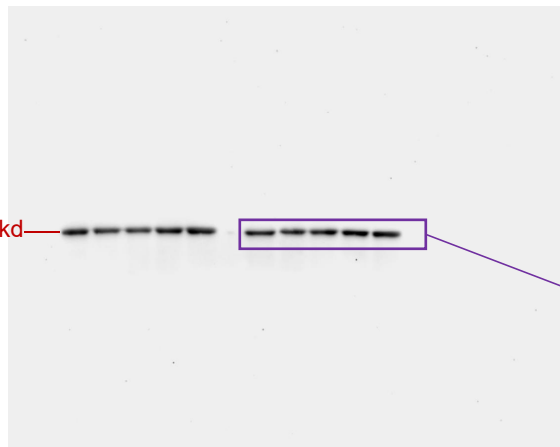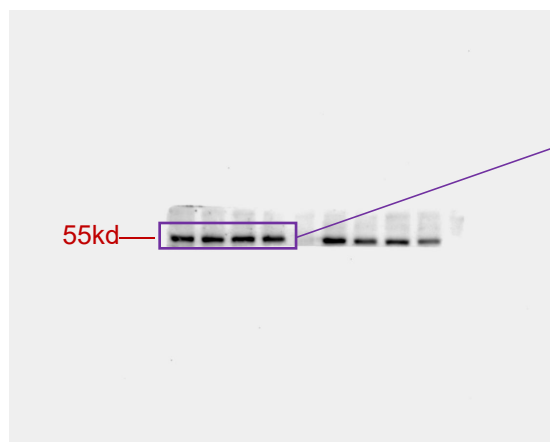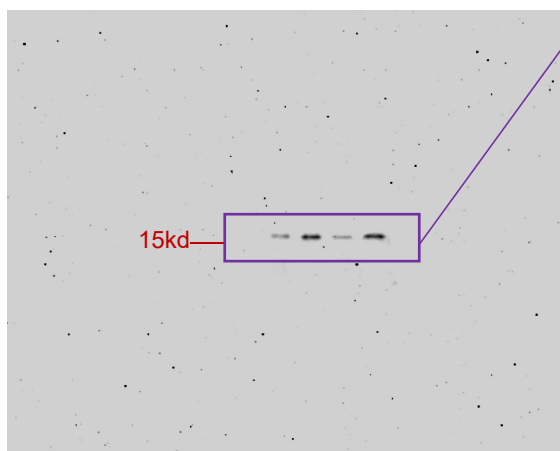

**A**

**Figure S1**

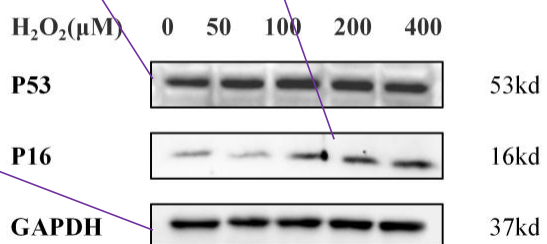

**C**

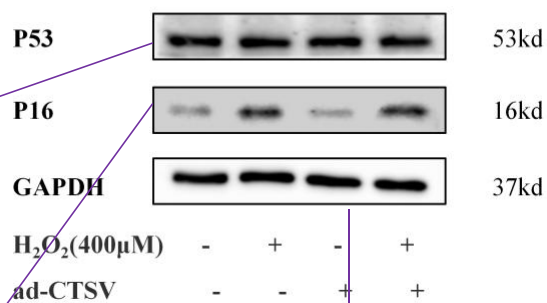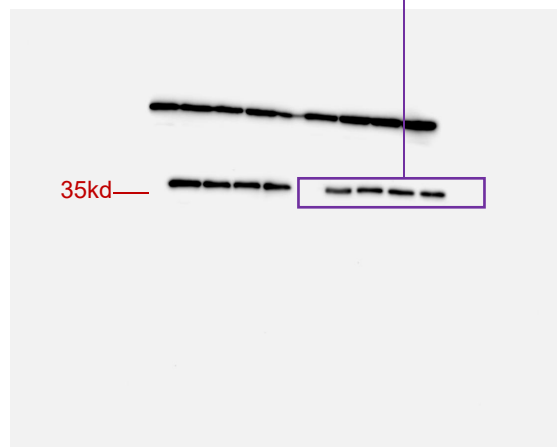

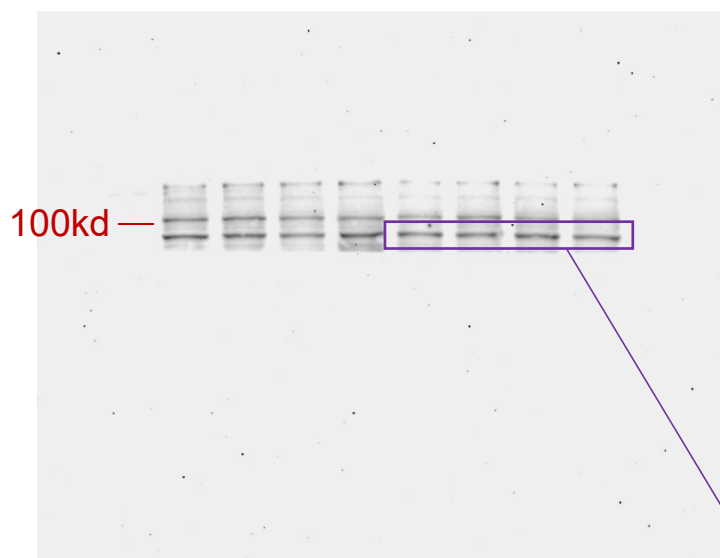

**Figure S3**

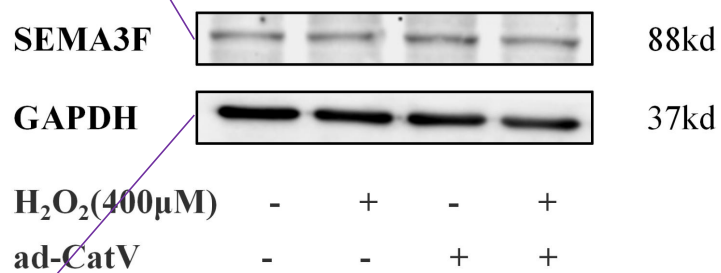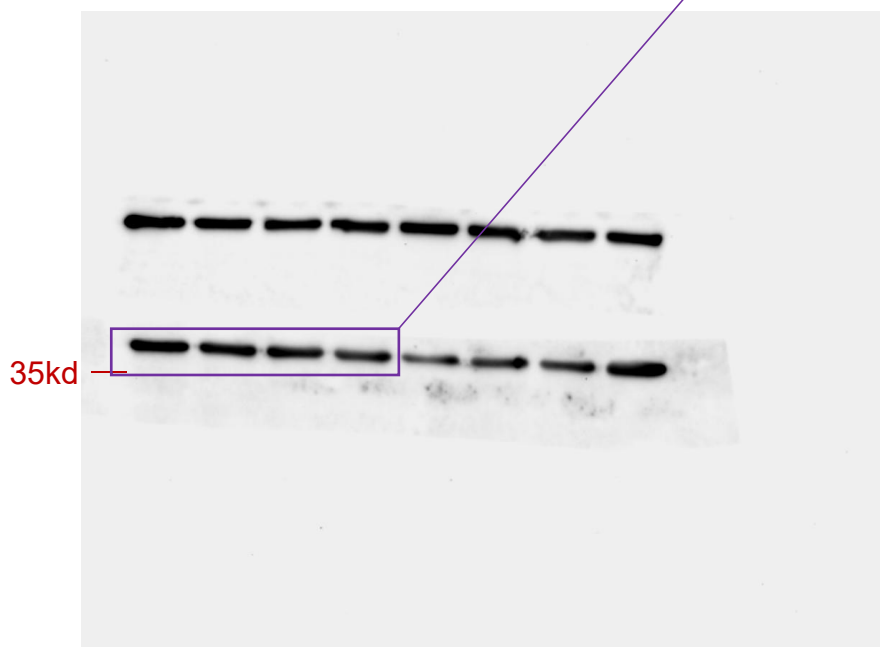

Supplement: Supplementary file 1 [file biology-12-00042-s001.zip › biology-2099799-WB.pdf]
